# Assessing live microbial therapeutic transmission

**DOI:** 10.1080/19490976.2024.2447836

**Published:** 2025-01-02

**Authors:** Jeremiah J. Faith

**Affiliations:** a Precision Immunology Institute, Icahn School of Medicine at Mount Sinai, New York, NY, USA; b Icahn Genomics Institute, Icahn School of Medicine at Mount Sinai, New York, NY, USA

**Keywords:** Microbiome, strain tracking, microbial therapeutics, fecal microbiota transplantation, live biotherapeutic product

## Abstract

The development of fecal microbiota transplantation and defined live biotherapeutic products for the treatment of human disease has been an empirically driven process yielding a notable success of approved drugs for the treatment of recurrent *Clostridioides difficile* infection. Assessing the potential of this therapeutic modality in other indications with mixed clinical results would benefit from consistent quantitative frameworks to characterize drug potency and composition and to assess the impact of dose and composition on the frequency and duration of strain engraftment. Monitoring these drug properties and engraftment outcomes would help identify minimally sufficient sets of microbial strains to treat disease and provide insights into the intersection between microbial function and host physiology. Broad and correct usage of strain detection methods is essential to this advancement. This article describes strain detection approaches, where they are best applied, what data they require, and clinical trial designs that are best suited to their application.

## Introduction

Fecal microbiota transplantation (FMT), consisting of a complex mixture of microbes from a fecal slurry, and live biotherapeutic products (LBP), composed of defined consortia of in vitro manufactured microbes, represent a new class of living microbial drug.^1,2^ These live microbial therapeutics have demonstrated broad success with two approved products for the treatment of recurrent C. difficile infection (rCDI).^3–6^ Beyond rCDI, live microbial therapeutics have shown promise for the treatment of primary C. difficile infection^7,8^ and a range of other diseases including ulcerative colitis,^9–15^ immunotherapy-related colitis,^16,17^ cancer,^18–20^ and decolonization of antibiotic resistant microbes.^21–24^ The clinical advancement of live microbial therapeutics requires demonstrating significant patient benefit through the identification of the relevant target population, endpoints, recruitment, and trial design including optional pretreatment with antibiotics or bowel lavage to aid the colonization of the microbes and dosing regimens that provide sufficient loads of microbes at sufficient frequency to provide the desired benefit to the host. These efforts have benefited from the robustness of *C. difficile* infection to variation in donor microbiota, dose, administration route, and manufacturing method as well as from continuous improvements in manufacturing to improve scale and reduce patient burden.^3,25–34^ However, the ideal future of live microbial therapies is to provide a defined consortium of the most effective microbial strains at the optimal dose and formulation to treat or prevent disease. To progress toward this goal of more precisely designed microbial therapeutics, the field needs to consistently use and improve tools for the characterization of FMT and LBP drug composition and potency in the drug product before it is administered and in the recipient following administration. Although numerous cross-sectional and prospective cohorts have enabled the identification of microbes associated with human disease, live microbial therapeutics represent the best opportunity to identify causal relationships between gut microbial strain engraftment and host physiology in humans. The foundational elements of such an exploration are to 1) have a functionally useful definition of a strain and 2) be able to track each strain across space and time. While early assessments have differed on whether higher strain engraftment is predictive of clinical response to FMT,^35,36^ collection of strain-level engraftment data across microbial therapeutic trials across diseases combined with clinical and basic science readouts of host physiology and pathology could identify if total strain engraftment, specific strain colonization, and transferred functional properties drive a given host response or if properties other than strain transmission are better predictors of response (e.g., transient influxes of microbe associated molecular patterns).

## What is a bacterial strain?

The observation that different genetic variants of a species have varied pathogenetic potential fueled the fields of microbial pathogenesis and infectious disease, enabling cross-strain comparative studies to identify toxins and other pathogenic elements that could also be tracked to identify and track disease outbreaks and to understand molecular mechanisms that facilitate new therapies and biotechnologies. The advancement of next-generation sequencing allowed the genomes of multiple pathogenic isolates to be sequenced from individual outbreaks.^37^

Broad genomic differences between bacterial isolates identify different strains that are unlikely to have evolved from a recent common ancestor, while strains with near identical genomes found across multiple infected individuals allow for the identification of the outbreak strain.^37–40^ Importantly, even within the outbreak strain, small genomic variations will occur over time and between hosts. Tracking these genetic variants can provide information on the order of infection spread and routes of transmission. Early work also demonstrated that metagenomic sequencing reads mapped to a sequenced pathogen strain can identify strain-specific genetic variants to cluster multiple samples from different individuals and different body sites by their relatedness to the pathogen.^41^ This approach represented an example of strain tracking a sequenced bacterial strain across numerous metagenomes and demonstrated that a reservoir for *Escherichia coli* and *Klebsiella pneumonia* blood stream infections is commonly the gut of the same individual.^41^

**Figure 1.**
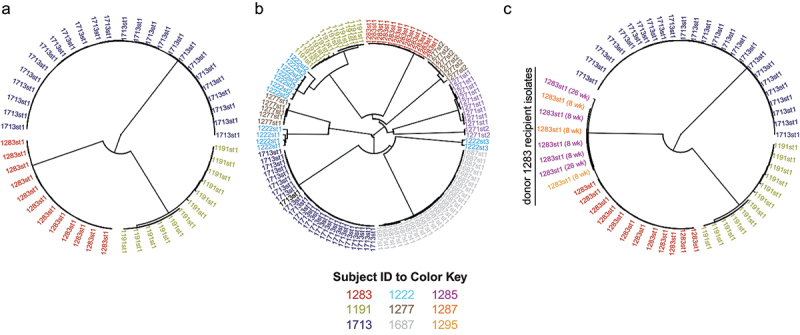
Natural genetic barriers within a species enable the tracking of strains. (a) Sequenced bacterial isolates from the same species and the same individual form a very tight cluster within a human with deep branches separating different subjects (shown examples are *Bifidobacterium adolescentis*; label text is strain name; label color indicates each subject). Replicate isolates typically vary by less than 0.04 in their genome kmer content. (b) Occasionally, individual humans harbor multiple strains of the same species. For example, subject 1222 has three different strains of *B. adolescentis* (labelled 1222st1, 1222st2, and 1222st3). These distinct strains cluster separately with deep branches like different strains of bacteria harbored in different individuals. (c) When isolates from different individuals cluster together, it typically indicates a transmission event of the strain. In the example, B. adolescentis strain 1283st1 from subject 1283 was isolated from three different FMT recipients (subjects 1285, 1287, and 1295) of subject 1283 8 to 26 weeks after the FMT procedure. *B. adolescentis* isolates for these analyses were previously isolated and sequenced ^50^. The distance between each strain was calculated as proportion of overlapping 20 nucleotide kmers between each pair of isolates. Trees were generated by complete-linkage hierarchical clustering.

Although a pathogenic strain provides a functional enrichment of medical interest that increases the chance that the same strain will be isolated and sequenced from multiple individuals, there are far more nonpathogenic strains than pathogenic ones, and these non- pathogenic strains are likewise transmitted throughout the human population to impact host physiology in myriad ways.^42^ As demonstrated by pathogen strain tracking, 1) two isolates of the same bacterial strain in an individual are far more similar than two random isolates of a species from different individuals, so they can be easily distinguished from their genomes; and 2) isolates of the same strain, even if isolated from the same individual, will likely have a few small nucleotide variants that distinguish them from each other. From these observations, a strain represents a collection of all isolates with near identical genome sequence that are a distinct cloud of genomic drift within the much broader cloud of genetic drift across a species.^43–47^ Large numbers of isolate genomes available for multiple species have enabled quantitative definitions of this concept. For example, dozens of sequenced isolates of the same species from the gut microbiota of one human yield one to three distinct genetic clouds with all isolates from a given cloud highly distinct from the other clouds (Figure 1(a)). If the same isolation and genome sequencing is done from the same human over time, the new isolate genomes almost always cluster tightly with one of the distinct clouds from the prior sample. If instead the same isolation and genome sequencing is performed from stool of a different human, new genetic clouds will be observed.^47–49^ Pairwise comparisons of genetic overlap between isolates in the same strain cloud typically find at least 96–98% genetic overlap in kmer content, where kmer is a short DNA sequence of length k (typically 20–40 nucleotides long).^48–50^ This empirical observation provides a genetic definition of a strain as isolates with genome sequences sharing >96% of their kmer content. For people with multiple strains of the same species in their gut microbiome, these strains are no more similar to each other than to strains in other people suggesting they do not evolve from the same original strain but instead are independently acquired and transmitted^50^ (Figure 1(b)). Strains isolated over time from the same individual typically have a similar functional impact on the host,^51^ despite their minor genetic differences. This natural clustering of strains within a species by genome alignment and kmer distance was also independently observed using an average nucleotide identity (ANI) metric establishing a cutoff of 99.5% ANI that was similar between both mammalian associated bacterial species and environmental species.^52^ Performing kmer overlap and ANI on the same set of bacterial genomes reveals that a threshold of 96–98% kmer overlap corresponds to approximately 99.8–99.9% ANI.^49,50^

Together, these studies provide a useful genomic definition of strains defined as sequenced bacterial isolates that share >96% kmer overlap or >99.8% ANI between their draft genomes with some flexibility for minor adjustments (96–98% kmer overlap, 99.5–99.9% ANI) depending on whether an analysis would be better served by a conservative (avoid false positives) or liberal (avoid false negatives) threshold. For the remainder of this discussion, we use these thresholds as the definition of a strain. The term strain is typically defined prior to use as it has several different meanings in the literature including as a synonym for “clone” (a genetically uniform population) and for describing pathogenic function in infectious disease. Given the variety of uses of the term strain, the genomic definition above has also be called a genomovar.^52^ For simplicity and consistency with prior literature, we will use the term strain.

## Approaches for detecting bacteria in a metagenome

The above definition of a strain as bacterial isolates with a shared genome kmer content of >96% and an ANI overlap of >99.8% provides a practical measure where any two isolates meeting this definition, in a context where a transmission was likely to occur (e.g., family members, FMT donor and recipient, infections in the same hospital; Figure 1(c)), are very likely recently derived from the same parent strain. Factors that limit tracking the recent source of a strain are the frequency of a strain in the human population and the total number of strains of a species that are in the human population. If a species has a few, very frequent strains that are transferred readily through the entire human population, it could be a challenge to identify its donor source in any transfer event. However, the gut microbiome has very few species with a small strain population size and high prevalence.^49^ Therefore, it should be possible to track strains from most species across thousands of recipients with little to no chance of finding the same strain by chance in two individuals.

### Species and genus tracking in desolate environments that are repopulated

**Figure 2.**
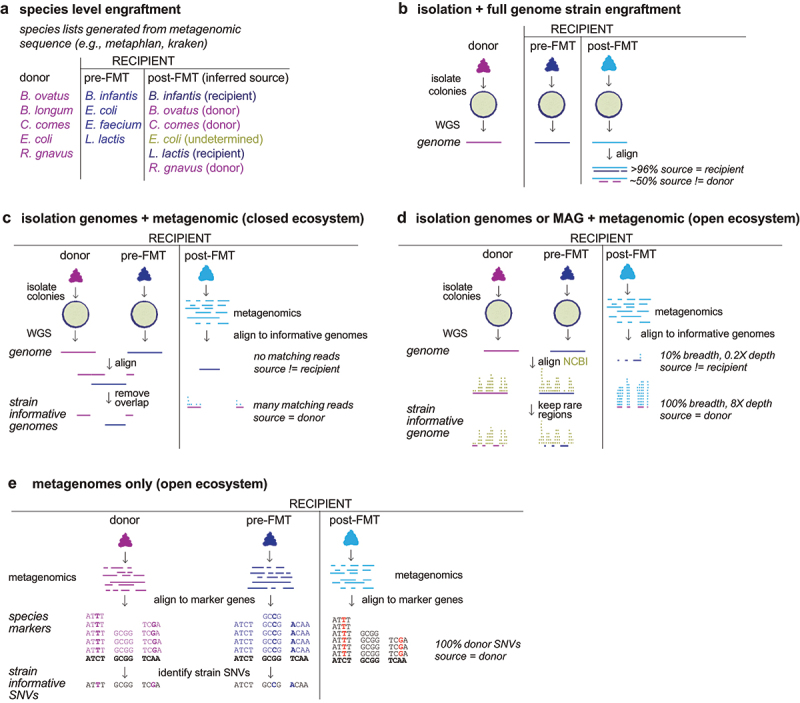
Varied approaches to track strain transmission. (a) Genus level (16S rRNA) or species level (metagenomics) taxonomic information can provide insights into strain transmission when the gut microbiome composition is highly varied between the donor and the recipient. In this context, donor taxa that are not in the recipient pre-fmt that are found in the recipient post-fmt are assumed to be transmitted from the donor. (b) Bacterial isolation with whole genome sequencing provides the highest confidence of a strain transmission. Whole genome sequences from all donor and recipient isolates are pairwise compared. Transmitted strains will have a kmer overlap of >96% and an ANI of >99.8. (c) In a closed ecosystem where genomes are available for all possible strains in the community, strain-specific abundances can be determined by first aligning all strain genomes to each other and removing genomic regions that are shared between any strains. The metagenomics samples can then be mapped to these strain informative genomes. Since the informative genomes have no shared genetic content, metagenomic reads mapping to a strain would indicate that the strain is present. Since the length of the informative content for a strain will depend on the uniqueness of its genome in the context of all other strains to be tracked, relative abundances can be scaled to the original genome size or a fixed genome size to account for this variation. (d) In an open ecosystem where genomes are available for the strains to be tracked but the complete set of possible strains in the target metagenomes is unknown, the informative region of each strain is determined by quantifying the frequency of each target strain region in the broader pangenome for that species (e.g., using all available genomes for the species in NCBI) and in a large set of metagenomes. The rarest 1–5% of each strain genome can serve as a strain-specific identifier. When the strain is present in a sample the strain’s sequencing depth with be significantly higher than that observed in negative control metagenomes and the relationship between the informative genome depth and breadth will follow a poisson distribution. (e) When only metagenomics data are available for FMT donors and recipients, strain-specific single nucleotide variants (snv)s for each species can be identified by mapping deeply sequenced metagenomes onto species marker genes or species reference genomes to identify regions of high coverage with a variant nucleotide compared to the reference. Metagenomes with a large number of overlapping SNVs for a given species are more likely to harbor the same bacterial strain.

In the context of extremely different ecosystems between a FMT donor microbiome and recipient microbiome, some analysis of microbial transmission in FMT can be performed using 16S rRNA amplicon sequencing and metagenomics sequencing with genus and species-level resolutions respectively. In the context of rCDI, where recipient microbiomes lack most of the taxa found in healthy subjects, identifying the species or genus overlap between the donor and post-FMT recipient, coupled with excluding the pre-FMT recipient taxa that overlap with the donor, provides insights into the transmission of the FMT drug organisms from the donor to the recipient (Figure 2(a)). In these studies, the recipient post-FMT microbiome, typically looks much more like the donor microbiome than the recipient pre-FMT microbiome.^53,54^ Although the recipient post-FMT microbiome also looks more like most FMT donors (and not just the microbiome of the donor they received), we would assume that an empty ecosystem given a large dose of a particular fecal microbiome (often 10^11^−10^13^ colony forming units [CFU]) is more likely to be colonized with the abundant donor strains than with the lower dose of other microbial strains they might be receiving from other environments. Therefore, the assumption is that these species or genera in the donor microbiome that newly arrive in the recipient post-FMT are strains transmitted from the donor. These analyses therefore can provide estimates of the proportion of the donor microbiome that is transferred as well as the particular taxa that are transferred.^53,55^

These species-based inferences of transmission are more challenging in FMT for indications without very distinct gut microbiome composition from a normal healthy adult, as the pre-FMT microbiome will likely have a large genus and species overlap with the donor microbiome, limiting the number of taxa that can be tracked. Even in the context of FMT for rCDI there are interpretation challenges with a species- or genus-level approach. First, the distinct microbiome in rCDI is in some part a reflection of the active antibiotic use in most subjects prior to FMT. It is therefore hard to know if any of the donor species are already in the recipient but are suppressed below the level of detection by the antibiotics. Second, strains in the recipient could also come from environmental sources beyond the donor. Overall, species and genus-level transmission analyses are the easiest to perform as they can use established user-friendly software,^56–59^ and they only require 16S rRNA amplicon sequencing or shallow metagenomics sequencing data.

### Genome-based strain tracking with cultured bacteria

The gold-standard strain tracking approach that best approximates Koch’s postulates^60,61^ is to culture the strain from the donor and determine its identity by genome sequencing (Figure 2(b)).^62^ The same strain should be cultured from the recipient post-FMT (but not pre-FMT) with identity verification by whole genome comparison between the two isolates. This approach is incredibly time intensive and not surprisingly has only been performed in a limited context.

Drewes et al. performed targeted culturing of potential pathobiont species from FMT donors and determined by genome sequencing that *Escherichia coli* isolated from the FMT donor stool could be isolated from the recipient post-FMT.^63^ Aggarwala et al. performed a broad high throughput culturing on five donor/recipient pairs and identified 48 donor strains that were reisolated from the recipients several weeks post-FMT^64^ (Figure 1(c)). This approach verifies that discrete culturable bacteria from the donor can be transferred by FMT and stably colonize the recipient. With entire genomic sequences of each strain available from the donor and recipient, the metric to identify the strain transmission is to simply compare the genome sequence of all the donor strains with the genome sequence of all the recipient strains and see if any of them have >96% kmer overlap or >99.8% average nucleotide identity (ANI). However, this culture-intense approach is not scalable to hundreds of donor and recipient samples with current technology, and it is limited to the culturable fraction of the microbiome in each sample.

### Genome-based metagenomic strain tracking in a completely defined (closed) ecosystem

A metagenome contains fragments of genomic content, typically from multiple organisms, at highly varied relative abundance. Shared genomic regions are common amongst microbes, particularly when multiple strains from the same species might be in the same sample. In the context of a completely defined community where every possible strain that could be in the ecosystem is known and sequenced, individual strains can be detected, and their relative abundances estimated, using the non-intersecting content of their genomes. For example, if an *in vitro* culture has two different strains of *E. coli* that share 60% of their genomic content, the shared content can be removed or masked from each genome, allowing the strains to be distinguished from each other using their remaining unique genomic content (Figure 2(c)). The cost of such masking is a reduction in sensitivity, since fewer metagenomic reads will map to the truncated target genomes. The non-unique regions can be ignored and relative abundances scaled according to the unique genome size of each strain^65^ or reads to non-unique regions can be assigned in proportion to the unique-region relative abundance of the strains the reads map to.^66–69^ As community size grows to include more strains and species, the available proportion of each genome that is unique will decrease. However, unless very similar strains are being tracked, there will likely be more than sufficient unique regions for tracking strains. A benefit of these closed ecosystem algorithms is that they are relatively straightforward to use. They only require the genome sequences of the isolates to be tracked and the metagenomes to track them in to estimate strain-specific relative abundances.

Although these known reference genome approaches are scalable and can accurately determine relative abundance, their application is limited to experimental scenarios where all potential community members are known, such as *in vitro* cultures and gnotobiotic mice. These algorithms do not typically have a statistical approach to determine the presence or absence of a given strain. However, a minimum relative abundance threshold could be used to call a strain present or absent with the relevant threshold estimated with a negative control dataset. These methods are not well suited for scenarios where only part of the community is known, as the reference genomes cannot be masked to remove overlapping regions between strains if not all of the strains are known.

### Genome-based metagenomic strain tracking in an undefined (open) ecosystem

In an open ecosystem where the target strains to track might be present but would be so in the context of an unknown number of other potential strains from diverse species, quantifying strain engraftment and abundance is similar to that of a closed ecosystem. Namely, the key task is still to identify the unique regions of each strain genome that allows the strain to be specifically detected in the context of other organisms. Unlike in a closed ecosystem where the identification of informative strain-specific genomic regions is completely defined, in an open ecosystem, we do not know what additional microbes might be present. Therefore, genomic regions are prioritized by identifying how rare each region (typically kmers of a specific length) is across thousands of bacterial genomes and metagenomes. The rarest genomic regions can then be used as strain-specific identifiers for quantifying engraftment and abundance (Figure 2(d)). The utility of this approach for a strain in a given species will depend on the structure of the species’ pangenome, with more open pangenomes having more rare strain-unique regions to identify the strain. Performance will also depend on the availability of genomes or metagenomes similar enough to the target strain to quantify the rarity of each region in a genome. For open ecosystem strain tracking, less of the strain genome will typically be used for detection than in the closed ecosystem scenario above, as tracking only the rarest 1–5% of the genome will limit false positives. However, this approach is still quite sensitive as only a proportion of each genome needs to be observed at less than 1X sequencing depth to detect and quantify each strain. A strain can be assessed present/absent by its observed sequencing depth in a given sample relative to that in a large set of negative control samples (i.e., samples where the strain are assumed to not be present)^64^ or by the sequencing depth and breadth of a metagenome across an informative strain genome relative to the expected relationship between depth and breadth that follows a Poisson distribution.^70–72^

This genome-based open ecosystem approach can sensitively track multiple discrete strains of the same species across metagenomes with unknown composition. It is therefore useful in the context of defined live biotherapeutic products where an in vitro manufactured community of bacteria is administered to one or more human recipients with undefined strain- level metagenomic composition.^70,73–78^ Given this approach’s sensitivity and precision and the tendency of FMT biobanks to use a limited number of FMT donors whose gut microbiome is largely stable over time, it can also be advantageous to perform high throughput culturing and genome sequencing of bacterial isolates from frequently used donors to track their abundance across many recipients.^64^ Any identified strains of potential therapeutic benefit are then also available for manufacturing scale-up in defined consortia with known transmission rates estimated in humans from FMT studies. These methods can also be used for tracking human- specific species genomes obtained from metagenomic assembly (MAGs) in contexts where no culturing is performed^79,80^ or to complement cultured genome tracking with MAGs of uncultured strains.^81^ MAGs could be generated from one or more donor samples, recipient samples prior to FMT or LBP administration, and recipient samples after FMT administration. From deep metagenomics datasets, these MAGs should capture many of the uncultured abundant strains in the donors and recipients. MAGs that overlap with cultured strain genomes can be eliminated. Limitations to using MAGs are that they will only capture the most abundant organisms in a metagenome and the quality of the assembly is lower than that of an isolate genome making it more susceptible to contigs contaminated from multiple organisms and other assembly noise.

These incorrect regions are highly likely to not exist in existing sequencing databases, and their rareness in such databases makes them a likely (but incorrect) choice as an informative genome region for strain tracking.

Despite the reduced sensitivity of this approach relative to the algorithms described for completely defined ecosystems, this type of algorithm is perhaps even better suited to quantify strain engraftment even in completely defined ecosystems as they have an established method to establish strain detection (present/absent) and are less susceptible to providing erroneous values if samples are mislabeled or experiments (in vitro or gnotobiotic) are contaminated. The disadvantage of this approach is the extra algorithm training required in a strain-specific manner to identify the rare regions of each genome and the need for genome sequences to track the organisms of interest. For defined LBPs, genome sequencies will almost certainly be available but they are less frequently available for FMT therapeutics.

### Metagenome-only strain tracking

With reduced costs in DNA sequencing, it is commonplace for microbiome studies to incorporate deep (>10 M paired-end reads) metagenomic sequencing as the assay for assessing microbiome composition. Given the ease of metagenomic sequence generation and its broad availability in published studies, methods of strain tracking that require only metagenomic data have a wide utility to improve our strain-level understanding of the microbiome. A key challenge of such methods is inferring the strains within each metagenome.

In the genome-based strain detection methods described above, the entire DNA sequence of a strain is available as a linked single unit such that finding a metagenomic fragment that maps to a unique sequence of a given strain genome increases the confidence that a strain is present. In metagenomics-only approaches, strains are inferred by mapping metagenomics onto species marker genes or a species-level reference genome database.^35,36,82–89^ With sufficient sequencing depth, single-nucleotide variations (SNVs) can be identified in the marker genes or in the reference genomes. The overlap in SNVs between two samples can be used to infer if the same strain might be in two different metagenome samples with more overlapping SNVs increasing the confidence that both samples contain the same strain (Figure 2(e)). The thresholds for assessing if SNV overlap is sufficient to conclude two samples have the same strain can be determined by simulation or by taking advantage of the known stability of strains in humans over time and the rarity of strain sharing between unrelated individuals. Collecting pairs of longitudinal samples from individuals sampled over time allows assessment of the overlap distribution in SNVs between likely true positives, whereas comparing metagenomic samples from two unrelated individuals enables the inference of a distribution of true negative SNV overlaps.^35,85^

Compared to genome-based strain tracking methods, metagenomics-only approaches have the advantage of far broader utility to take advantage of the wealth of available metagenomes to compare thousands of samples and make inferences about strain sharing in the context of FMT and beyond. However, the lack of a known genome from an isolated organism for tracking leads to several limitations. First, the strains are inferred from SNVs relative to a reference marker gene or genome. To confidently infer an SNV requires several- fold sequence coverage at a given SNV (e.g., 10X). Therefore, very deep metagenomic sequencing is required to detect lower abundance organisms (e.g., 10X coverage of a genome at 0.1% would require 300 million 150nt paired-end reads), so the strain comparisons likely have poor sensitivity for taxa at <1% relative abundance. Secondly, it is more challenging to interpret the results describing the sharing of strains inferred from a metagenome, as no strain genome or cultured isolate is available. Finally, it is an unresolved challenge to infer how many strains of a species are in a metagenome. Although metagenomics approaches can infer when species vary in polymorphic complexity in metagenomes, suggesting that some species may harbor more than one strain in a given individual,^84,90,91^ the precise determination of the number of strains in a species in a microbiome is currently best done experimentally.^50^ Therefore, the tracking of strains via metagenomics-only approaches typically focus on the determination of a single dominant or composite strain per species, although newer methods attempt to overcome this limitation. In practice, many species in the gut typically have only one or two strains per human host,^47,50^ so this type of strain tracking still has great utility.

## Next steps in improving the performance and utilization of strain tracking approaches

Unlike broadly utilized bioinformatics tools for searching DNA sequencing databases, mapping RNA-seq reads, or exploring scRNA-seq datasets, the tools for bacterial strain tracking are still relatively niche research tools whose use requires setting numerous thresholds, building custom sequence databases and other hurdles that limit their broader use. These research tools have improved dramatically in their short development times but to improve adoption of strain detection approaches, future software packages should be simple to install with good default databases and thresholds for calling a strain present/absent in a metagenome based on its genome sequence, as well as reliable strain inference in metagenomics-only approaches.

Minimum program inputs would provide a set of genomes and metagenomes for genome-based trackers or only a set of metagenomes for metagenome-only strain trackers. Minimum outputs would be a table with each strain, its detection status in each sample, a confidence estimate of the detection, and the relative abundance of the strain if detected. Existing and new algorithms would benefit from performance comparisons on standardized large test datasets of true positive isolate genomes with paired deep metagenomes as well as standardized large training datasets of longitudinal samples where strain sharing over time can be inferred and contrasted to the background signal in unrelated individuals. Comparing MAGs with metagenomically inferred strain SNVs would provide insights into if MAGs from deeply sequenced FMT donor metagenomes combined with genome-based trackers provide increased sensitivity compared to metagenomic SNV strain-inference that benefits from deep sequencing on all samples.

The development or utilization of new experimental technologies could dramatically improve our ability to identify and detect strains in metagenomic samples. Hi-C,^92^ deep long- read sequencing,^93,94^ or single-cell genome sequencing^95^ could improve strain detection while limiting false positives.

## Insights from strain tracking

Although genus-level 16S rRNA amplicon sequencing and species-level metagenomics provide insights into broad changes in community composition in the context of microbial therapeutics, implementation of strain tracking approaches can provided more specific insights to identify strain engraftments associated with response/non-response and to inform the design of future defined consortia. In the context of a strongly altered microbial ecosystem such as in rCDI, it may be that the number of microbes engrafting to restore basic microbiome function is as important as the types of organisms colonizing. Strain tracking methods allow for assessment of which strains in the donor engraft in the recipient microbiome and for how long. Traditional metrics like alpha diversity post-FMT can identify if responding individuals have more even and richer community composition than non-responders, while with strain tracking you can quantify if the number of donor strains added or the number of recipient strains removed by the therapy is higher in responders vs non-responders. Strain tracking will also allow the determination of whether strains initially colonize all subjects but do not durably engraft in relapsing patients.

For many applications, it will likely be important to determine not just whether more donor strain engraftment is predictive of success, but also which strains or strain functions are most associated with clinical success upon engraftment. To best quantify the association of strain engraftment with clinical endpoints, it is important to have many replicates of the same strains therapeutically administered to different recipients. Since the gut microbiome of healthy individuals is largely stable over time, FMT from the same donor is largely composed of the same set of strains.^48,64^ Therefore, trial designs that use few FMT donors for many different recipients provide more opportunities to estimate statistical associations than single donors into single recipients.^15^ LBP trials typically transplant one defined community of bacteria into many recipients providing a clear example where the engraftment of specific strains can be associated with trial outcomes.

Measuring the impact of drug dose and frequency on subsequent drug levels in the recipient as well as the impact of a specific drug level on host physiology is a fundamental property of drug development and clinical trial design. A trial with insufficient dosing of the active compound could falsely conclude a beneficial drug was ineffective. Unlike antibody and small molecule drugs, strains in microbial therapeutics are alive and typically replicating. If microbial therapeutic strains are dosed at a high enough initial potency in a host with an available niche for their future growth, the strains will continue to replicate and durably maintain colonization in the host for weeks to years. As a community, we will hopefully gain sufficient understanding of microbial function that we can understand which microbes need to colonize which recipients to confer a specific health benefit. Although we are likely far from such understanding in a deep mechanistic way across subjects with different diseases that respond to FMT, improved study of the transmission and colonization duration of therapeutically administered strains will provide practical insights into minimal sets of strains sufficient to treat a given disease and what dose and frequency is required to maintain these beneficial strains. Likewise, identifying minimally sufficient strain groupings necessary for therapeutic benefit could help narrow down the search for understanding the mechanism of action for these therapies. Although the FDA-approval of two stool-based live microbial therapeutics for the treatment of rCDI is a great achievement for the microbiome field, we have a long road ahead to understand indications that benefit from live microbial therapeutics, optimal dosing, drug mechanism of action, and factors driving response or non-response. FMT trials commonly do not quantify drug potency or strain composition, while both FMT and defined LBP trials often do not quantify the strain-specific engraftment of the drug in the recipient. While we may continue to stumble our way to clinical success with this approach, characterizing the potency and composition of these drugs combined with tracking their engraftment frequency and duration in recipients as standard practice for clinical trials using existing tools such as those described above would provide immediate practical insights to better understand completed trials and to better design future ones.
